# Multi-species probiotic supplement enhances vagal nerve function – results of a randomized controlled trial in patients with depression and healthy controls

**DOI:** 10.1080/19490976.2025.2492377

**Published:** 2025-04-29

**Authors:** Sabrina Mörkl, Martin Narrath, Daria Schlotmann, Marie-Therese Sallmutter, Julia Putz, Julia Lang, Andreas Brandstätter, Rene Pilz, Helmut Karl Lackner, Nandu Goswami, Bianca Steuber, Jasmin Tatzer, Sonja Lackner, Sandra Holasek, Annamaria Painold, Emanuel Jauk, Julian Wenninger, Angela Horvath, Nicolai Spicher, Asmus Barth, Mary I Butler, Jolana Wagner-Skacel

**Affiliations:** a Division of Medical Psychology, Psychosomatics and Psychotherapeutic Medicine, Medical University of Graz, Graz, Austria; b Division of Physiology und Pathophysiology, Medical University of Graz, Graz, Austria; c Gravitational Physiology and Medicine Research Unit, Division of Physiology und Pathophysiology, Medical University of Graz, Graz, Austria; d Center for Space and Aviation Health, College of Medicine, Mohammed Bin Rashid University of Medicine and Health Sciences, Dubai, United Arab Emirates; e Division of Immunology, Medical University of Graz, Graz, Austria; f Division of Psychiatry and Psychotherapeutic Medicine, Medical University of Graz, Graz, Austria; g Division of Gastroenterology and Hepatology, Medical University of Graz, Graz, Austria; h Department of Medical Informatics, University Medical Center Göttingen, Göttingen, Germany; i Department of Psychiatry and Neurobehavioural Science, University College Cork, Cork, Ireland

**Keywords:** Vagus nerve, probiotic, depression, heart rate variability, gut microbiome

## Abstract

Major depression (MD) significantly impacts individual well-being and society. The vagus nerve plays a pivotal role in the gut-brain axis, facilitating bidirectional communication between these systems. Recent meta-analyses suggest potential antidepressant effects of probiotics, although their mechanisms remain unclear. This study aimed to assess the impact of a multi-species probiotic (OMNi-BiOTiC® STRESS Repair) on vagus nerve function in 43 MD patients and 43 healthy controls (HC). Participants received either probiotics or placebo twice daily. Serum and stool samples were collected at baseline, 7 days, 28 days, and 3 months. Vagus nerve (VN) function was evaluated using 24-hour electrocardiography (ECG) for heart rate variability (HRV), alongside stool microbiome analysis via 16S rRNA sequencing. After 3 months, MD patients receiving probiotics demonstrated significantly improved morning VN function compared to HC. MD participants who were in the probiotic group showed a significant increase in *Christensellales*, particularly *Akkermansia muciniphila* along with improved sleep parameters (use of sleep medication, sleep latency) as measured by the Pittsburgh Sleep Quality Inventory (PSI). This study highlights potential physiological benefits of probiotics in MD, potentially mediated through VN stimulation. Understanding these mechanisms could lead to novel therapeutic approaches for MD management.

## GRAPHICAL ABSTRACT

**Figure:**
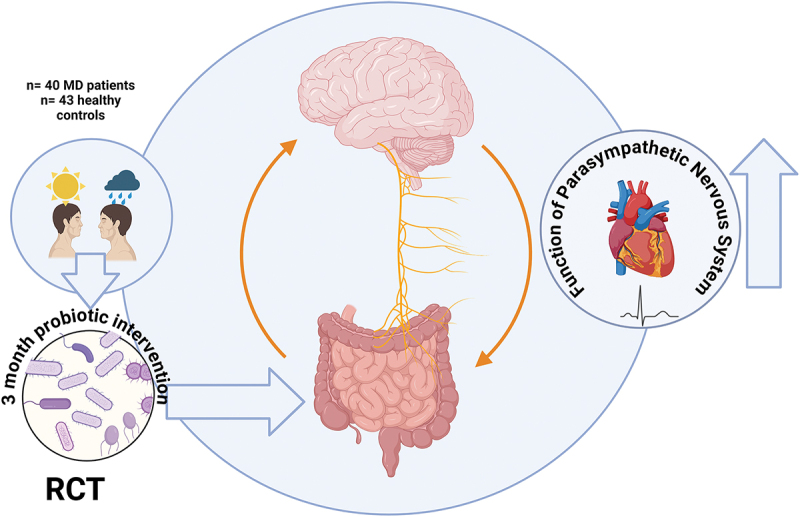

## Introduction

The microbiota-gut-brain axis (MGBA) facilitates bi-directional communication between the gut and brain, primarily via the vagus nerve (VN) which is composed of 80% afferent and 20% efferent fibers.^1^ The VN senses microbiota and metabolites, transmitting information to the CNS. Additionally, VN function involves a cholinergic anti-inflammatory pathway, reducing inflammation and intestinal permeability.^1^

Patients with major depression (MD) show distinct gut microbiota compositions, higher gut permeability, and elevated inflammatory markers.^2^ Studies like the Flemish gut flora project have linked gut microbiome features with depression, identifying specific microbial profiles associated with quality of life indicators.^3^

VN function, measured by heart rate variability (HRV), is altered in depression,^4,5^ with reduced HRV correlating with greater depression severity. HRV depicts cardiac autonomic regulation and refers to the degree of fluctuation in the length of the interbeat (R-R) intervals obtained from an electrocardiography (ECG). Essentially, a higher HRV (higher VN function) indicates better general health.^6^ A meta-analysis based on data from 673 MD participants and 407 healthy controls (HC) revealed that MD patients showed significantly reduced HRV parameters compared to HC and the severity of depression was negatively correlated with HRV (*r* = −.35).^7,8^ In a previous study of our group, we showed that gut microbiome diversity correlated with VN function and inflammatory parameters. Butyrate producers such as *Ruminococcus* and *Faecalibacterium* were more abundant in participants with better VN function.^9^

Probiotics have been demonstrated to improve depressive symptoms and are recommended as an adjunct treatment for depression.^10^ However, human studies exploring the effects of probiotics on the gut microbiome and VN function are limited. A pilot study investigating the effect of *Lactobacillus reuteri* in veterans with post-traumatic stress disorder (PTSD) showed significantly decreased mean heart rate of the probiotic group compared to the placebo group^11,12^; however, traditional HRV parameters were not measured. Another placebo-controlled trial by Romao da Silva et al., included 40 women with hypertension receiving either a multispecies probiotic or a placebo, suggested an improvement in autonomic modulation.^13^ Our study assessed the impact of a multispecies probiotic (OMNi-BiOTiC® STRESS Repair) on vagus nerve function in MD patients and HCs. To our knowledge, this is the first human study to describe the gut microbiota together with long-term HRV parameters in MD. We hypothesize that a multispecies probiotic supplementation as an adjunctive treatment for MD can stimulate VN function, leading to increased VN activity through alterations in the gut microbiota.

## Materials and methods

### Study design

**Figure 1.**
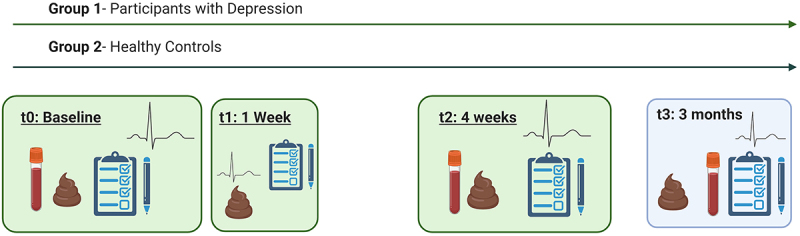
Study schedule. Participants underwent blood and stool sampling, heart rate variability measurements and questionnaires at baseline, after 12 weeks and at 3 months. After one week stool sampling, questionnaires and heart rate variability measurement were performed.

This pilot, monocentric, randomized, placebo-controlled trial was conducted at the Department of Psychiatry and Psychotherapeutic Medicine at the Medical University of Graz, Austria. Patients with MD (diagnosed by the M.I.N.I.-International Neuropsychiatric Interview) and HC were randomized into two groups: probiotic and placebo. Serum and stool samples, clinical variables, 24-hr HRV, and questionnaires were collected at baseline, 7 days, 28 days, and 3 months. The study schedule is shown in Figure 1. This study was registered at *clinicaltrials.gov* (NCT-04772664) on 16^th^ August, 2021 and approved by the ethics review board (EK-No: 33–227 ex 20/21). All participants provided written informed consent. The primary endpoint of this study was the change in VN function, assessed by HRV. Secondary endpoints included changes in depressive symptoms, measured by the Beck Depression Inventory (BDI) and the Hamilton Depression Rating Scale (HAMD), as well as improvements in sleep quality and changes of gut microbiome composition. These endpoints were selected to evaluate the impact of the probiotic intervention on VN function and its potential effects on mood and sleep.

### Participants

Participants were recruited at the Department for Psychiatry and Psychotherapeutic Medicine, or via the online recruitment-service Probando (*www.probando.io*.). Inclusion criteria were written informed consent, age 18–65 years, and for MD patients, an MD-diagnosis per International Classification of Diseases, 10th Revision (ICD-10) criteria. All patients met these criteria, and diagnoses were confirmed through clinical assessments conducted by experienced psychiatrists.

Exclusion criteria included acute suicidality, lack of consent, known cardiovascular disorders, pregnancy or breastfeeding, severe active drug dependence, severe mental or cerebral diseases, severe head trauma or brain surgery, active tumors, mental disability, dementia, severe autoimmune diseases or immunosuppression, recent antibiotic therapy, chronic laxative abuse, acute infectious diarrhea, gastrointestinal surgery (except appendectomy), and regular probiotic supplement use in the past year. Participants could not take supplements, probiotics, or prebiotics during the trial. Antibiotic or pre- or probiotic use during the trial resulted in drop-out.

### Probiotic intervention

Individuals in the probiotic group received a multispecies probiotic (OMNi-BiOTiC® -STRESS Repair) for 3 months (1 sachet, 3 g twice daily, 1.5 × 10^10^ CFU in total) from Allergosan®, Graz, Austria. This probiotic contains 9 bacterial species: *Bifidobacterium bifidum W23, Bifidobacterium lactis W51, Bifidobacterium lactis W52, Lactobacillus acidophilus W22, Lactobacillus casei W56, Lactobacillus paracasei W20, Lactobacillus plantarum W62, Lactobacillus salivarius W24*, and *Lactococcus lactis W19*, with at least 7.5 billion microorganisms per sachet. These species were selected for their anti-inflammatory properties^14^ and effects on reducing cognitive reactivity to sad mood,^15^ as well as VN activation observed in animal models.^16–19^ The placebo group received identical sachets in color, consistency, and taste, containing maize starch, maltodextrin, inulin, potassium chloride, magnesium-sulfate, fructooligosaccharides, amylases, and manganese-sulfate. Both probiotics and placebos were stirred in water and consumed twice daily (morning and evening). The study was double-blind. Participants were required to adhere to the intervention, avoid additional probiotics, and maintain their current diet, lifestyle and physical activity. MD patients continued their usual psychopharmacological treatment and standard care.

### Questionnaires

The M.I.N.I. was conducted by an experienced psychiatrist to exclude HC with any psychiatric diagnosis and confirm MD diagnoses, including secondary diagnoses. Depressive symptoms were assessed using the Hamilton Depression Scale (HAMD)^20^ and the Beck Depression Inventory-II (BDI).^21^ Additional questionnaires included the Pittsburgh Sleep Quality Inventory (PSQI)^22^ for sleep quality, the Leids-R^23^ for cognitive reactivity, and the Perceived Stress Scale (PSS)^24^for stress levels.

Clinical and demographic data (age, weight, height, BMI, sex, smoking, medication) were documented at each time point. Dietary intake was monitored with the Vienna food record^25^ (24-hour-recalls) at baseline and follow-ups. Physical activity was assessed with the International Physical Activity Questionnaire (IPAQ).^25^ These questionnaires were completed at four time points.

### Heart rate variability (HRV)

Continuous ECG were out with a portable device (eMotion Faros 180^TM^, Einthoven Lead II set-up; weight: 13 g). Raw data were converted from EDF to MATLAB® data format for further analysis. To check the quality of the physiological data and calculate the inter-beat interval time series, we have been using a semi-automatic artifact-handling device developed by our research group.^26^

In brief, its main criteria are (a) the pattern of the QRS complex and the time of occurrence within the ECG to identify “ectopic beats”, (b) physiological limits on an individual, age-depending basis, and (c) the maximal percentage of change in relation to the standard deviation of the signal.^27^

For the calculation of the variables, 120 s intervals within the 24-hour measurements were chosen in order to ensure the stability and stationarity of the signal, considering that the segment should be no less than approximately ten times the wavelength of the lower frequency bound of the investigated component.

All HRV variables were calculated following the recommended guidelines.^28,29^ The time domain HRV parameters were SDNN (standard deviation of the regular R-R intervals [[normal-to-normal]; NN]), RMSSD (square root of the mean squared differences of successive R-R intervals), pNN50 (proportion of NN50 divided by the total number of NN intervals), and logRSA (log-transformed value of respiratory sinus arrhythmia).^30^ While the RMSSD, pNN50, and logRSA are influenced more by VN function, the SDNN represents the activity of both sympathetic and parasympathetic branches. All HRV variables were computed in MATLAB® (Version 9.9, The MathWorks, Inc., Natick, Massachusetts, United States, 2020), except for the logRSA which was computed by a self-developed algorithm following the definition in Moser et al.^30^

For the frequency domain variables, Power Spectral Density (PSD) estimates were calculated from the R‐R intervals via Burg’s method (model order 24) after removing the trend (2^nd^‐order). Low frequency (LF) was defined as 0.04–0.15 hz, high frequency (HF) was defined as 0.15 hz −0.40 hz. Due to the skewed distribution of frequency domain variables, a natural logarithmic transformation was applied.

Average values of the 120s intervals of the HRV variables for 24 h, morning (9:00–12:00 am), afternoon (1:00–4:00 pm), restful and restless sleep were used for further analysis.^27^

### Gut microbiome analysis

Microbiome analysis was conducted at the Center for Medical Research (ZMF, Medical University of Graz). One-gram stool samples were stored at −80°C. Sequence analysis followed supplier recommendations, using Illumina MiSeq as detailed by Klymiuk et al.^31^ DNA extraction was performed with the QIAsymphony DSP Virus/Pathogen Kit (Qiagen, Germany). The hypervariable V3–V4 regions of the bacterial 16S rRNA gene were amplified from fecal DNA using target-specific primers and the FastStart High Fidelity PCR system (Sigma, Germany). PCR reactions (25 µl) included initial denaturation at 95°C for 3 minutes, followed by 30 cycles of 95°C for 45 sec, 55°C for 45 sec, 72°C for one minute, and a final elongation at 72°C for seven minutes. Amplification products were quantified, pooled, normalized, indexed, purified, and sequenced at the ZMF Core Facility Molecular Biology using an Illumina MiSeq desktop sequencer with v3 chemistry and 600 cycles (2 × 300).

### Analysis of microbiome data

Sequences underwent quality assessment using the FASTQ tool. Paired-end reads were pre-filtered (quality threshold > 28), trimmed, and filtered for quality and chimeras using the DADA2 library in R.^32^ Taxonomic assignments were conducted with DADA2 against the SILVA SSURef database,^33^ following recommended parameters from the DADA2 manual. Operational taxonomic units (OTUs) that were not identified at the genus level and those that occurred in 10% or fewer samples were excluded from the analysis. Bacterial taxonomic diversity was estimated using different diversity-indices. Differential abundance analysis was performed with Linear Discriminant Analysis Effect Size (LEfSe)^34^using the R package microbiomeMarker or Microbiome Multivariable Association with Linear Models (MaAsLin) using the maaslin2 package. Principal coordinate analysis based on either weighted or unweighted UniFrac, Jaccard or Bray-Curtis dissimilarities was conducted and dispersion and cetroid patterns were analyzed by PERMANOVA using the packages phyloseq and vegan in R. In addition, redundancy analysis was performed on Hellinger-transformed abundance data using the vegan package in R.

### Statistical evaluation, sample size, randomization and visualization

Analyses were conducted in SPSS Version 23.0 (BM Corp., Armonk, NY, USA), and data visualization used QIIME^35^ outputs and GraphPad Prism 6 (Graph Pad Software, San Diego, CA, USA). All data are presented as mean and standard deviation unless specified otherwise. Normal distribution was checked using the Kolmogorov–Smirnov test.

A power analysis was conducted using G*Power 3.1. In the category of F-tests, the “Repeated Measures ANOVA within-between interaction” procedure was selected. Assuming a small effect size of 0.2, an alpha level of 0.05, and 95% power, a total sample size of 80 participants (20 participants per group) is required for the study, accounting for four groups (HC: Probiotic/Placebo; MD: Probiotic/Placebo).

Randomization was conducted using www.randomization.com (block randomization) by the manufacturer of both the probiotic and placebo. The manufacturer prepared the product packages according to the randomization list provided by the person in charge of the process, with group assignments designated for each study participant. Each participant received three packages, one for each month of treatment, and these were labeled with the corresponding participant code. The packages could only be distinguished by this code, ensuring blinding. Both the person responsible for randomization and all unblinded employees at the manufacturer were sworn to confidentiality. Group allocation followed a 1:1 ratio for probiotic and placebo groups. The entire study team remained blinded to the randomization until the study’s conclusion.

Between group differences were compared with t-tests, u-tests, or X^2^ tests depending on meeting normality assumptions. To investigate the effects of interest, a repeated measures ANOVA was used to evaluate the effects of the between-subjects factors probiotic supplementation (Probiotic: yes vs. no) and diagnosis type (Diagnosis: MD vs. HC) on domains across time points. Time point of measurement was specified as a within-subjects factor (Time: 4 levels). Homogeneity of variances and sphericity were tested using Mauchly’s test, with Greenhouse-Geisser corrections for any violations. All tests were two-tailed, with *p* < .05 considered significant.

## Results

### Participants

**Figure 2.**
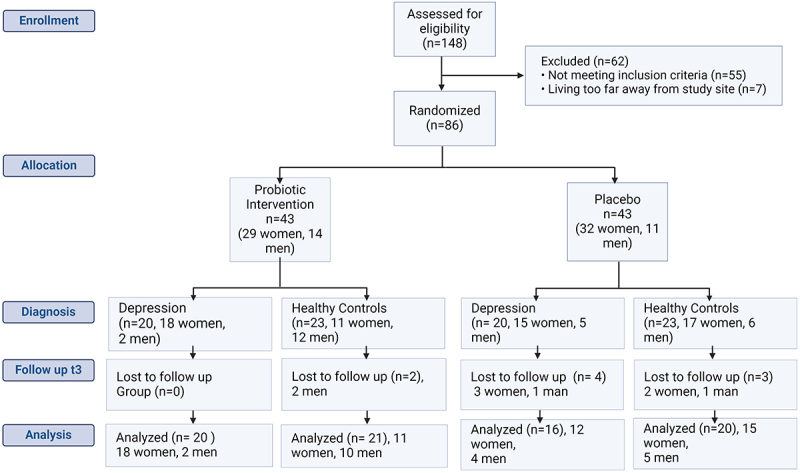
Consort flow diagram.

The CONSORT Flow Diagram summarizes the participant enrollment (Figure 2). One hundred and forty-eight persons were assessed for eligibility, whereas 62 were excluded as they did not meet the inclusion criteria (*n* = 55) or were living too far away from the study site. The study population was comparable in their main characteristics, except for significantly more smokers in the MD group (Table 1, Supplementary Table S1).

**Table 1.** Baseline characteristics.

	Probiotic Group			Placebo Group		
	Depression (*n* = 20)	Healthy Control (*n* = 23)	p-value	Depression (*n* = 20)	Healthy Control (*n* = 23)	*p*-value
Sex (female)	16	11	0.029	15	17	.935
smokers	6	4	0.329	8	2	.015
	mean (SD)	mean (SD)		mean (SD)	mean (SD)	
Age (years)	32.65 (8.83)	35.30 (10.10)	0.407	37.5 (14.73)	37.13 (14.68)	.884
Weight [kg]	75.00 (18.12)	68.32 (14.22)	0.183	72.35 (20.51)	67.98 (10.97)	0.383
Height [m]	1.69 (0.84)	1.71 (0.92)	0.578	1.69 (0.96)	1.70 (0.90)	0.828
BMI [kg/m^2^]	26.00 (5.95)	23.14 (3.26)	0.108	24.99 (6.66)	23.36 (3.33)	0.441
RR sys [mmHg]	120.70 (15.93)	124.86 (13.25)	0.354	122.25 (15.34)	128.74 (11.87)	0.126
RR dia [mmHg]	78.20 (11.43)	81.39 (8.11)	0.293	78.80 (10.29)	82.56 (9.71)	0.225
Pulse [bpm]	77.20 (12.24)	69.13 (10.87)	0.027	75.85 (10.04)	70.26 (9.40)	0.067
BDI (t0)	17.16 (11.68)	3.95 (4.39)	<0.001	21.89 (10.62)	3.87 (3.44)	<.001
HAMD (t0)	18.10 (10.91)	2.05 (1.40)	<0.001	18.42 (9.44)	1.70 (1.55)	<.001
PSS Score (t0)	31.45 (7.31)	21.62 (5.53)	<0.001	34.94 (6.14)	20.45 (5.37)	<.001

BDI = beck depression inventory; HAMD = hamilton depression scale, BMI = body mass index. RR = Riva Rocci, blood pressure; sys = systole; dia = diastole; bpm = beats per minute. PSS = perceived stress scale.

### HRV

#### 24hrs HRV measurements

**Figure 3.**
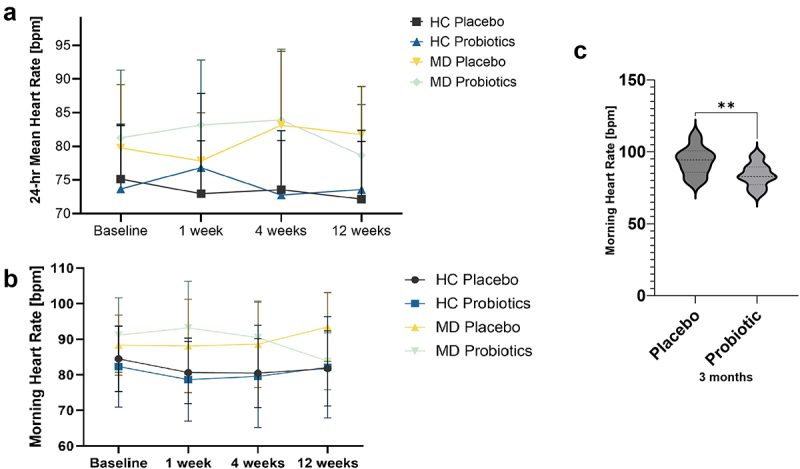
24-hr heart rate (a) and morning heart rate (b) at 4 time points for healthy controls (HC) and patients with depression (MD), and morning heart rate for depressive patients (MD) at 3 months comparing the probiotic and placebo groups (c). Statistical comparisons were performed using a repeated-measures ANOVA for panels a and b, and an unpaired Student’s t-test for panel c. Sample sizes: (a, b baseline) *n* = 21 (HC placebo) *n* = 22 (HC probiotic) *n* = 17 (MD placebo) *n* = 20 (MD probiotic); (c, after 3 months) *n* = 35, *n* = 15 (placebo), *n* = 20 (probiotic). bpm = beats per minute.

Regarding the main effect of the between-subjects factor diagnosis, depressive patients showed significantly higher heart rate in comparison to HC (*F*_(1, 66)_ = 19.01, *p* < .001, *η^2^* = .22; Figure 3a).

The repeated measures ANOVA for 24-hour meanHR revealed a significant interaction between Time and Probiotic, (*F*_(3, 198)_ = 3.24, *p* = .027, *η^2^* = .05), as well as between Time and Diagnosis, (*F*_(3, 195)_ = 4.35, *p* = .021, *η^2^* = .05).

For all participants, after one week, those taking a probiotic had significantly lower heart rate than those taking a placebo (*t*_(71.21)_ = −2.18, *p* = .033), while at the other time points there were no significant differences. For patients and for HC separately, there were no significant differences at the four time points between the probiotic and placebo group.

MD patients receiving a probiotic had significant decreases in mean HR from time point 2–4 (*t*_(17)_ = 2.48, *p* = .024, *d* = −0.62, 95%-KI [−1.17, −0.06,]) and 3–4 (*t*_(18)_ = 2.12, *p* = .048, *d* = 0.14, 95%-KI [−0.35, 0.63]), while MD patients receiving a probiotic had significant increases in mean heart rate from time point 2–4 (*t*_(17)_ = 2.48, *p* = .024, *d* = −0.62, 95%-KI [−1.17, −0.06]) and time point 3–4 (*t*_(18)_ = 2.12, *p* = .048, *d* = 0.14, 95%-KI [−0.35, 0.63]).

Regarding pNN50, RMSSD and SDNN over 24 hours, there was a significant effect for Diagnosis (*F*_(1, 65)_ = 6.87, *p* = .011, *η^2^* = 0.09, *F*_(1, 66)_ = 9.94, *p* = .002, *η^2^* = 0.13 and *F*_(1,66)_ = 14.26, *p* < .001, *η^2^* = 0.18, respectively), whereby MD patients showed significantly lower values than HC. However, there was no significant difference between the probiotic and placebo or over the time points. Further, there were no significant differences regarding lnHF. Concerning 24hrs logRSA, repeated measures ANOVA revealed a significant interaction between time and diagnosis (*F*_(3, 198)_ = 2.79, *p* = .042, η^2^ = .04) but not for probiotic intake. There was a significant decrease of logRSA between time point 2 and 3 *t*_(16)_ = 3.67, *p* = .002, *d* = 0.89, 95%-KI [0.32, 1.45] and a significant increase between time point 3 and 4 in MD participants *t*_(18)_ = −2.14, *p* = .047, *d* = −0.49, 95%-KI [−0.96, −0.01].

#### Morning and afternoon HRV measurements

##### Heart rate

For heart rate in the morning, here was a significant difference between MD and HC, (*F*_(1,64)_ = 9.72, *p* = .003, *η^2^* = 0.11), whereby patients showed significantly higher heart rate in comparison to HC (Figure 3b).

Heart rate in the morning revealed a significant interaction of Time and Probiotic intake (*F*_(3, 192)_ = 3.99, *p* = .009, *η^2^* = .06), and a Time × Probiotic × Diagnosis interaction (*F*_(3, 192)_ = 6.06, *p* < .001, *η^2^* = .09).

MD patients taking a probiotic had significantly lower heart rate after three months of probiotic intake in comparison to those taking a placebo (t_(33)_ = 3.23, *p* = .003, *d* = 1.10, 95%-KI [0.38, 1.82] (Figure 3c).

For the afternoon values, heart rate revealed no significant interactions for Time, but a significant between subject effect of Diagnosis (*F*_(1,64)_ = 11.72, *p* = .001, *η^2^* =.15), with patients having significantly higher heart rate than HC.

##### Vagus parameters: pNN50, RMSSD, lnHF, logRSA

There was a significant difference between participants with MD and HC regarding pNN50 in the morning (*F*_(1,64)_ = 7.18, *p* = .009, *η^2^* = .10) and in the afternoon (*F*_(1,64)_ = 6.60, *p* = .012, *η^2^* = .09), with HC having significantly higher pNN50. For pNN50 in the morning, the ANOVA revealed a trend of an interaction between Time × Probiotic × Diagnosis (*F*_(3, 192)_ = 2.63, *p* = .052, *η^2^* = 0.04).

MD patients had lower RMSSD in the morning (*F*_(1,64)_ = 9.92, *p* = .004, *η^2^* =.13) and in the afternoon (*F*_(1,66)_ = 7.80, *p* = .007, *η*^2^  = .11)in comparison to HC.

**Figure 4.**
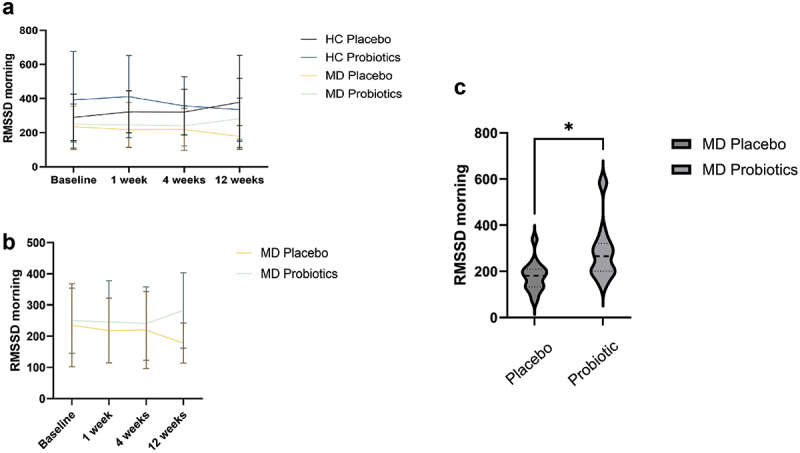
RMSSD morning values at 4 time points for healthy controls (HC) and patients with depression (MD), comparing the probiotic and placebo groups (a), for the MD group only (b), and RMSSD differences for patients with MD after 3 months of taking a probiotic (c). Statistical comparisons were performed using a repeated-measures ANOVA for panels a and b, and a Mann-Whitney U-test for panel c. Sample sizes: (a, b, baseline) *n* = 21 (HC placebo), *n* = 22 (HC probiotic), *n* = 17 (MD placebo), *n* = 20 (MD probiotic); (c) *n* = 35, *n* = 15 (placebo), *n* = 20 (probiotic). RMSSD = root mean square of successive differences.

RMSSD in the morning showed a significant interaction effect of Time × Probiotic × Diagnosis (*F*_(3, 192)_ = 4.18, *p* = .007, *η^2^* = .06) (Figure 4a,b). MD patients taking a probiotic showed significantly higher RMSSD values in the morning compared to those taking a placebo after 3 months of probiotic intake (*U* = 241.00, *Z* = 3.03, *p* = .002; Figure 4c). There was a significant increase of RMSSD in patients taking a probiotic between time point 3 and time point 4 *t*_(19)_ = 2.70, *p* = .014, *d* = 0.60, 95%-KI [0.12, 1.07].

lnHF in the morning (F_(1,64)_ = 6.80, *p* = .011, *η^2^* = .09) and in the afternoon (*F*_(1,66)_ = 6.39, *p* = .014, *η^2^* = .08) was significantly higher in HC than in MD patients (*F*_(1,64)_ = 6.80, *p* = .011, *η* ^2^ =.09).

**Figure 5.**
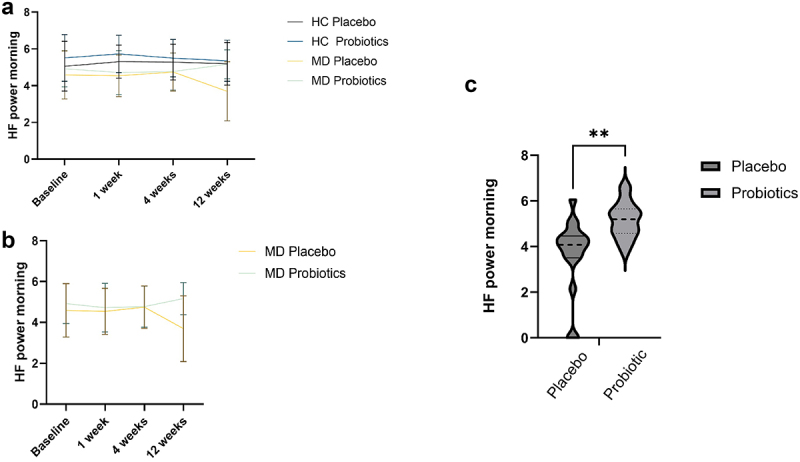
High-frequency (HF) power morning values at 4 time points for healthy controls (HC) and patients with depression (MD), comparing the probiotic and placebo groups (a), for the MD group only (b), and HF power differences for patients with MD after 3 months of taking a probiotic (c). Statistical comparisons were performed using a repeated-measures ANOVA for panels a and b, and an unpaired Student’s t-test for panel c. Sample sizes: (a, b, baseline) *n* = 21 (HC placebo), *n* = 22 (HC probiotic), *n* = 17 (MD placebo), *n* = 20 (MD probiotic); (c) *n* = 35, *n* = 15 (placebo), *n* = 20 (probiotic). HF = high frequency.

Further, lnHF in the morning showed a significant interaction of Time × Probiotic × Diagnosis (*F*_(2.726, 192)_ = 2.99, *p* = .037, *η^2^* = .02; Figure 5a,b). There was a significant difference in lnHF at three months between MD patients receiving a placebo and patients receiving a probiotic (*t*_(33)_ = −3.48, *p* = .001, *d*= −1.19, 95%-KI [−1.91, −0.45]), with the probiotic group having significantly higher lnHF scores than the placebo group (Figure 5c). MD patients taking the probiotic had a significant increase in lnHF from time point 3 to time point 4 (*t*_(19)_ = −2.48, *p* = .022, *d* = 0.39 (95%-KI [0.06, 0.72]).

MD patients had significantly lower logRSA in the morning (*F*_(1,64)_ = 8.799, *p*= .004, *η^2^* = .12) and in the afternoon (*F*_(1,66)_ = 8.860, *p*= .004, *η^2^* =.12) compared to HC.

**Figure 6.**
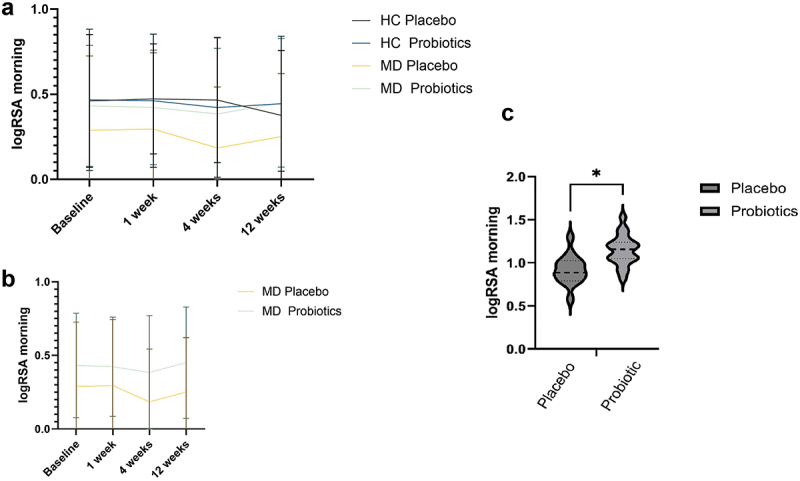
Logarithm of respiratory sinus arrhythmia (LogRSA) morning values at 4 time points for healthy controls (HC) and patients with depression (MD), comparing the probiotic and placebo groups (a), for the MD group only (b), and LogRSA differences for patients with MD after 3 months of taking a probiotic (c). Statistical comparisons were performed using a repeated-measures ANOVA for panels a and b, and an unpaired Student’s t-test for panel c. Sample sizes: (a, b, baseline) *n* = 21 (HC placebo), *n* = 22 (HC probiotic), *n* = 17 (MD placebo), *n* = 20 (MD probiotic); (C) *n* = 35, *n* = 15 (placebo), *n* = 20 (probiotic). LogRSA = logarithm of respiratory sinus arrhythmia.

Regarding logRSA in the morning, there was a significant interaction effect of Time × Probiotic × Diagnosis (*F*_(3, 192)_ = 3.74, *p* = .012, *η^2^* = .06; Figure 6a,b). After three months, MD patients receiving probiotics had significantly higher logRSA in the morning than MD patients receiving a placebo (*t*_(33)_ = −3.88, *p* < .001, *d* = 1.33, 95%-KI [0.58, 2.06], Figure 6c). MD patients receiving a probiotics had a significant increase in morning logRSA from baseline to three months *t*_(18)_ = −2.30, *p* = .033, *d*=-0.53, 95%-KI [−1.00, −0.04] and from 4 weeks to three months *t*_(19)_ = −3.01, *p* = .007, *d*=-0.67, 95%-KI [−1.15, −0.18].

##### SDNN

**Figure 7.**
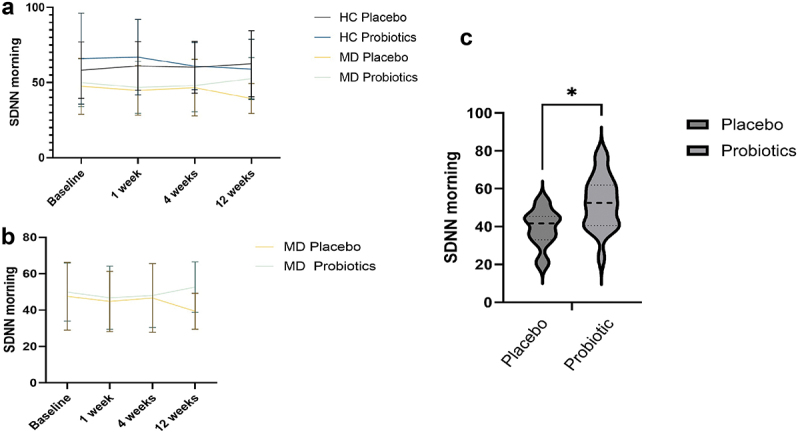
Standard Deviation of the NN intervals (SDNN) morning values at 4 time points for healthy controls (HC) and patients with depression (MD), comparing the probiotic and placebo groups (a), for the MD group only (b), and SDNN differences for patients with MD after 3 months of taking a probiotic (c). Statistical comparisons were performed using a repeated-measures ANOVA for panels a and b, and an unpaired Student’s t-test for panel c. Sample sizes: (a, b, baseline) *n* = 21 (HC placebo), *n* = 22 (HC probiotic), *n* = 17 (MD placebo), *n* = 20 (MD probiotic); (c) *n* = 35, *n* = 15 (placebo), *n* = 20 (probiotic). SDNN =  Standard Deviation of the NN intervals.

SDNN was significantly lower in MD patients compared to controls for morning (F_(1,64)_ = 11.609, *p* = .001, η^2^  = 0.15) and for afternoon values (F_(1,66)_ = 11.295, *p*=.001, *η^2^* = 0.15). For morning SDNN values, there was a significant interaction effect of Time*Probiotic*Diagnosis (F_(3, 192)_ = 3.543, *p* = .016, *η*^2^  = .052; Figure 7a,b). MD patients taking a probiotic had significantly higher SDNN after three months compared to those taking a placebo (*t*_(33)_ = −3.142, *p* = .004, Cohens *d*= −1.073, KI [−1.78, −0.35]; Figure 7c).

#### HRV during sleep

MD participants had significantly higher heart rate during restful sleep (F_(1,68)_ = 16.479, *p*=.001, *η*^2^ =.19) and restless sleep (F_(1,68)_ = 13.402, *p* =.001, *η^2^* =.17), the same was found for pnn50, with MD patients having significantly lower values for restful sleep (F_(1,68)_ = 8.301, *p* = .006, *η^2^* = .09) and restless sleep (F_(1,68)_ = 8.204, *p* = .006, *η^2^* = .10), regardless of taking a probiotic. There was a significant interaction of Time * Diagnosis (F_(3, 66)_ = 3.094, *p* = .028, *η^2^* = .044) for pnn50 during restful sleep. MD participants had a significant increase in pnn50 during restful sleep between time point 3 and 4 (*t*_(33)_ = −2.78, *p* = .009, *d* = −0.48, 95%-KI [−0.83, −0.12]).

With regard to RMSSD in restful sleep, there was a significant interaction of Time*Diagnosis (F_(3, 204)_ = 3.075, *p* =.029, *η^2^* = .043). MD patients had significantly lower RMSSD in restful sleep (F_(1,68)_ = 6.753, *p*=.011, *η^2^* = .09) and in restless sleep (F_(1,68)_ = 9.155, *p*=.003, *η^2^* = .12). Patients with MD had a significant increase of RMSSD in restful sleep from time point 3 to 4 (*t*_(33)_ = −1.875, *p* = .070, *d* = −0.32, 95%-KI [−0.03, 0.66,]).

lnHF in restful (F_(1,68)_ = 6.919, *p*=.011, *η^2^* = .09) and restless sleep (F_(1,68)_ = 7.787, *p*=.007, *η^2^* = .10) was significantly lower in MD patients compared to HC. With regard to logRSA also significant differences for restless sleep (F_(1,68)_ = 8.204, *p* =.006, *η^2^ =* .11) and restful sleep (F_(1,68)_ = 8.031, *p* = .006, *η^2^ =* 0.11) between patients and controls could be found, regardless of probiotic intake.

SDNN in restful (F_(1,68)_ = 7.172, *p* =.009, *η^2^ =* .09) and restless sleep (F_(1,68)_ = 12.336, *p* < .001, *η^2^ =* .15), showed significant differences between MD and HC participants. There was also a significant interaction of Time*Diagnosis for SDNN during restful sleep (F_(3, 204)_ = 2.777, *p* =.049, *η^2^* = .039). MD participants showed a significant increase in SDNN during restful sleep from time point 3 to time point 4 (*t*_(33)_ = −2.42, *p* = .021, *d* = −0.41, [−0.76, −0.06]).

### Gut microbiome

A total of 313 stool samples were obtained (143 samples from patients with depression and 170 samples from healthy controls, 157 samples from participants receiving probiotics and 156 samples from participants receiving placebo), while there were samples available from 66/86 (76.74%) participants at all four time points.

#### Richness and diversity

Compared to HC, MD patients had significantly lower Chao-1 diversity (F_(1,62)_ = 6.094, *p*=.017, *η^2^ =* .11), number of observed species (F_(1,62)_ = 7.865, *p*=.007, *η^2^  = .11*), Simpson-Index (F_(1,62)_ = 5.119, *p*=.027, *η^2^  = .07*) and Shannon-Index (F_(1,62)_ = 9.555, *p*=.003, *η^2^  = .13*); however, diversity was not changed significantly by probiotic intake or over time.

**Figure 8.**
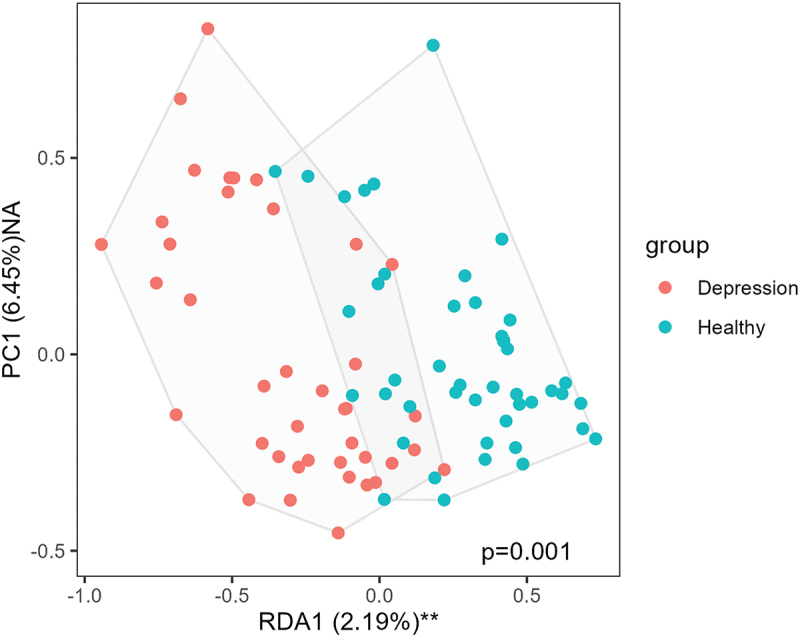
Difference in beta-diversity between patients with depression (MD, red dots) and healthy controls (HC, blue dots). Statistical comparisons were performed using redundancy analysis (RDA) with principal component (PC) scores for group differentiation. Sample sizes: *n* = 39 (MD), *n* = 44 (HC). RDA = redundancy analysis; PC = principal component.

There was a significant difference of beta-diversity at baseline between patients with depression and healthy controls (*p* = .001; Figure 8). PCoA and redundancy analysis did not show a significant effect of the probiotic on the overall microbiome composition over time.

#### Analysis of differentially abundant species

At baseline, patients showed significantly more abundance of *Blautia* and *Blautia subspecies*, while HC had higher abundance of butyrate producers such as *Prevotella, Facealibacterium, Coprococcus, Clostridia* and *Oscillospirales*.

There were no significant changes in the gut microbiome composition of MD patients due to the probiotic intervention. MaAsLin2 idendified one OTU (*Lactococcus lactis*) to be upregulated by the probiotic intervention in HC.

**Figure 9.**
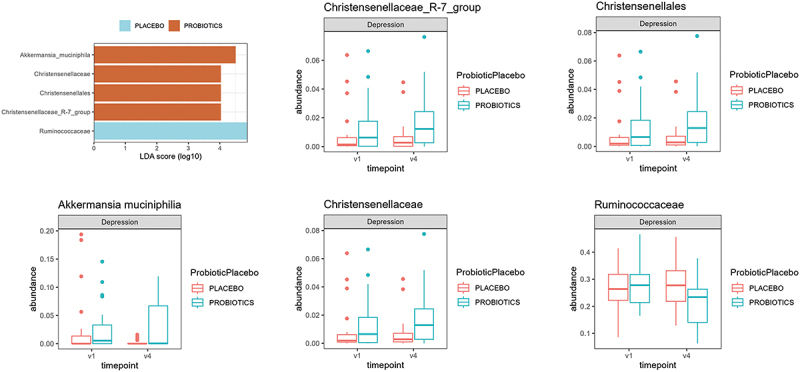
Differences in taxa between patients with depression taking a placebo and those taking a probiotic at time point 4 (12 weeks). Statistical comparisons were performed using differential abundance analysis. Sample sizes: *n* = 15 (placebo), *n* = 19 (probiotic).

Because we could see a significant impact of VN parameters at the 3 month timepoint in MD patients, we calculated microbiota differences with MaAsLin2 between timepoint one and timepoint four (3 months) in MD patients. *Christensenellales taxa (*including *Akkermansia muciniphila)* were higher and *Ruminococcaceae* were lower in MD after 12 weeks of probiotic intervention (Figure 9).

### Changes in clinical parameters/questionnaires

#### PSS- Stress Score

Regarding PSS-Score, there was a significant difference between MD patients and HC (F_(1,52)_ = 46.715, *p* < .001, *η^2^ =* .47). Also, there was a significant effect for Time (F_(2.605, 156)_ = 5.477, *p* = .002, *η* ^2^  = .09) and interaction effect of Time*Diagnosis (F_(2.605, 156)_ = 3.924, *p* = .014, *η^2^* = 0.070), signifying that the probiotic and placebo group decreased similarily in stress, while there was more improvement in the group with clinical depression, particularly from time point 1–2 (*t*_(28)_ = 3.17, *p* = .004, *d*= .59, [0.19, 0.98], time point 1–3 (*t*_(32)_ = 3.60, *p* = .001, *d* = 0.63, [0.25, 0.99]) and time point 1–4 (*t*_(29)_ = 3.98, *p* < .001, *d*= .73, [0.32, 1.13]).

#### Depression scores

MD patients differed significantly from HC (F_(1,60)_ = 58.746, *p* < .001, *η^2^ =* .49) regarding the BDI and HAMD scores (F_(1, 65)_ = 87.835, *p* < .001, *η^2^* = .58). Further, there was a significant effect of Time, indicating that study participants improved significantly during the course of the study regarding BDI scores (F_(2.520, 180)_ = 14.954, *p* < .001, *η* ^2^  = .20) and HAMD-scores (F_(2.520, 180)_ = 14.954, *p* < .001, *η^2^* = .20) irrespective of belonging to the probiotic or placebo group.

For the HAMD-score there was a significant interaction of Time and Diagnosis (F_(3, 195)_ = 7.743, *p* < .001, η^2^ = .11). MD patients decreased significantly in HAMD, particularly from time point 1–2 (*t*_(34)_ = 4.76, *p* < .001, *d* = .81, [0.42, 1.18]), time point 1–3 (*t*_(33)_ = 3.98, *p* < .001, *d* = .68, [0.31, 1.05]), and time point 1–4 (*t*_(34)_ = 3.91, *p* < .001, *d* = .66, [0.29, 1.02]).

Further, LEIDS-r total score results were significantly higher in MD compared to HC (F_(1,82)_ = 37.36, *p* < .001, *η^2^ = *.31). Additionally, there was a significant effect of Time (F_(2.526, 207.159)_ = 9.472, *p* < .001, *η^2^* = 0.10), and a significant between subject effect of Diagnosis (F_(1,82)_ = 37.353, *p* < .001, η^2^ =.31), as both groups improved significantly over time and MD patients improved more than HC irrespective of taking a probiotic.

Regarding LEIDS-r subscsales, MD patients scored significantly higher in terms of suicidality (F_(1, 57)_ = 53.752, *p* < .001, *η^2^* =.48), aggression (F_(1, 57)_ = 17.739, *p* < .001, *η^2^* = .24), risk aversion (F_(1, 57)_ = 41.814, *p* < .001, *η^2^* = .42) and rumination (F_(1,57)_ = 53.68,*p*<.001, *η^2^ =* .48).

For the subscale aggression, there was a significant interaction of Time and Probiotic (F_(3, 171)_ = 4.485, *p* =.005, *η^2^* = .07), and Time and Diagnosis (F_(3, 171)_ = 2.658 *p* = .050, η^2^ = 0.045). Those taking a probiotic, had significantly less aggression from time point 1–2 (*t*_(35)_ = 3.23, *p* = .003, *d* = 0.54), time point 1–3 (*t*_(37)_ = 2.54, *p* = .016, *d* = 0.41) and time point 1–4 (*t(34)* = 3.46, *p* = .001, *d* = 0.58).

Rumination significantly decreased over time (F_(2.596, 147.979)_ = 14.637, *p* < .001, *η*^*2*^ = .204). Further, there was a significant interaction of Time*Diagnosis (F_(2.596, 147.979)_ = 3.008, *p* = .039, *η*^*2*^ =.050),with MD participants experiencing significantly less rumination between time point 1–4 *t*_(31)_ = 2.91, *p* = .007, *d* = .51 and time point 3–4 *t*_(31)_ = 2.14, *p* = .041, *d* = .38. There was also a trend for interaction of Time and probiotic intake (F_(2.596, 147.979)_ = 2.704, *p* = .056,*η*^*2*^ = .045), with significant decreases in rumination for those receiving probiotics between time points 1–2 (*t*_(35)_ = 3.86, *p* < .001, *d* = .64), 1–3 (*t*_(37)_ = 3.62, *p* < .001, *d* = .59), 1–4 (*t*_(34)_ = 5.66, *p* < .001, *d* = .96), and 2–4 (*t*_(31)_ = 2.60, *p* = .014, *d* = .46).

#### PSQI

MD patients had significantly higher PSQI total scores compared to HC (F_(1,57)_ = 32.756, *p* < .001, *η^2^* = .36).

We found a significant interaction effect of Time and Probiotic (F_(2.554, 171)_ = 2.886, *p* =.046, η^2^ = .05) and a significant interaction effect of Time*Probiotic*Diagnosis (F_(2.554, 171)_ = 7.849, *p* =.046, η^2^ = .05).

**Figure 10.**
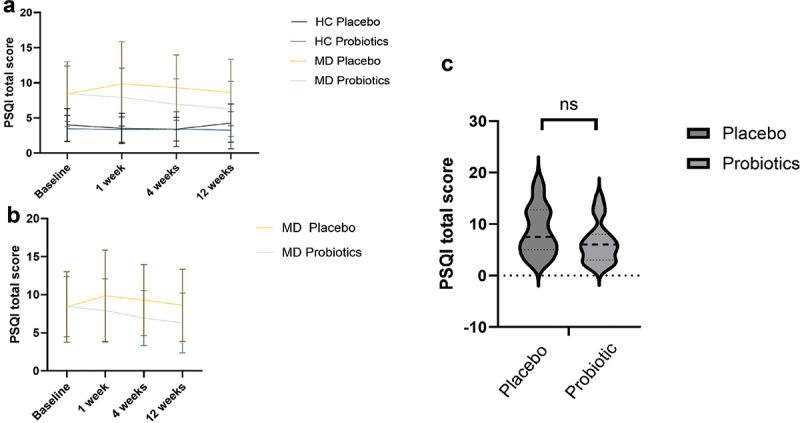
Pittsburgh Sleep Quality Inventory (PSQI) total scores at 4 time points for healthy controls (HC) and patients with depression (MD), comparing the probiotic and placebo groups (a), for the MD group only (b), and PSQI differences for patients with MD after 3 months of taking a probiotic (c). Statistical comparisons were performed using a repeated-measures ANOVA for panels a and b, and a Mann Whitney U-test for panel c. Sample sizes: (a, b, baseline) *n* = 23 (HC placebo), *n* = 21 (HC probiotic), *n* = 18 (MD placebo), *n* = 20 (MD probiotic) (c) *n* = 33, *n* = 16 (placebo), *n* = 17 (probiotic). PSQI = Pittsburgh Sleep Quality Inventory Score.

Participants taking a probiotic had significantly improved PSQI scores between time point 1–2 (*t*_(35)_ = 2.24, *p* = .032, *d* = .37), time point 1–3 (*t*_(37)_ = 2.60, *p* = .013, *d* = .42) and time point 1–4 (*t*_(34)_ = 2.56, *p* = .015, *d* = .43). MD patients receiving a probiotic had significantly improved PSQI scores between time point 1–3 (*t*_(17)_ = 2.90, *p* = .010, *d* = .68.), 1–4 (*t*_(16)_ = 3.21, *p* = .005, *d* = .78), 2–4 (*t*_(13)_ = 2.70, *p* = .018, *d* = .72). By trend but not statistically significant, participants taking a probiotic had lower PSQI scores than those taking a placebo after 3 months of probiotic intake (U = 832, *p*=.083) (see Figure 10a–c).

#### PSQI Subscores

In PSQI subscores, MD patients scored significantly worse in sleep quality (F_(1,54)_ = 16.66, *p* < .001, *η^2^* = .24), sleep latency (*F*_(1, 57)_ = 9.13, *p* = .004, *η^2^* = .14), sleep disturbances (*F*_(1, 59)_ = 10.77, *p* = .002, *η^2^* = .15), sleep efficacy (*F*_(1, 62)_ = 8.49, *p* = .005, = .12), daytime dysfunction (*F*_(1, 57)_ = 52.84, *p* < .001, *η^2^* = .48) and use of sleep medication (F_(1,57)_ = 24.543, *p* < .001, *η^2^* = 0.301). There were no significant differences between MD and HC regarding sleep duration.

Sleep quality was not affected by Time or Probiotic. Sleep latency showed a significant interaction effect of Time and Probiotic (F_(1,171)_ = 2.951, *p* =.034, *η^2^* = 0.049). Participants in the probiotic group needed significantly less time to fall asleep compared to the placebo group after 12 weeks (t_(68)_ = 2.141, *p* = .036, *d* = 0.512, 95% CI [0.034, 0.987]).

Sleep duration significantly changed over time (F_(3,171)_ = 2.942, *p* = 0.035, *η^2^* = 0.049), but there was no interaction effect with probiotic intake, but with Time*Diagnosis, as patients with MD had decreased sleep duration over time (F_(3,171)_ = 2.759, *p* = .044, *η^2^* = 0.046). Sleep efficacy was not significantly changing over time and was not affected by probiotic intake.

Regarding the use of sleep medication, there was a significant three way interaction of Time*Probiotic*Diagnosis (F_(3,123.002)_ = 3.388, *p* = .034, *η^2^* = 0.056). MD patients receiving a probiotic needed significantly less sleep medication from time point 2 to 3 (*t*_(15)_ = 2.06, *p* = .050, *d* = .51), and a trend toward less sleep medication between timepoint 2 to 4 (*t*_(13)_ = 1.85, *p* = .088, *d* = .49).

### Correlations

#### Alpha diversity and vagal parameters

Chao-1 diversity and number of observed species at baseline was negatively correlated to heart rate at baseline (*r* = −.29, *p* = .007) and (*r* = −0.29, *p* = .007); however, there were no further correlations with vagus parameters. Simpson-index at baseline was correlated to 24-hr-heart rate (*r* = −.24, *p* = .030), 24 hr RMSSD (*r* = .25, *p* = .022), heart rate in the morning (*r* = .31, *p* = .005), 24 hr-logRSA (*r* = .27, *p* = .014), 24-hr SDNN (*r* = 0.31, *p* = .004) and SDNN in the morning (*r* = .31, *p* = .006) at baseline but not at other time points. Shannon-index at baseline was correlated to mean 24 hr heart rate (*r* = −.25, *p* = .022), 24 hr RMSSD (*r* = .24, *p* = .032), heart rate in the morning (*r* = .24, *p* = .032), logRSA in the morning (*r* = 0.22, *p* = 0.047), 24 hr SDNN (*r* = 0.26, *p* = 0.019) and SDNN in the morning (*r* = 0.22, *p* = 0.046).

#### Alpha diversity with scores of questionnaires

Chao-1 diversity was negatively correlated to PSQI-score at baseline (*r* = −.26, *p* = .019) and PSS at baseline (*r* = −.32, *p* = .004). After 1 week, there were still negative correlations to PSQI score (*r* = −0.24, *p* = .036) and PSS (*r* = −.28, *p* = .041) and also after 4 weeks a correlation with Chao-1 and PSQI score was seen (*r* = −.27, *p* = .024).

## Discussion

This study is the first to demonstrate that a multi-species probiotic can significantly alter VN parameters and gut microbiome composition in patients with MD. These changes were significant for morning VN measurements after three months of probiotic intake. We also confirmed earlier findings of gut microbiome differences between MD patients and HC.

After three months, MD participants who were in the probiotic group showed a significant increase in *Christensellales*, particularly *Akkermansia muciniphila* along with improved sleep paramters (sleep latency, use of sleep medication) as measured by the PSQI. In summary, these results suggest that both probiotic intervention and diagnostic status may have significant effects on VN function.

Prior research has indicated that probiotics affect the VN. Romao da Silva et al. found distinct VN function changes in 40 women with hypertension after a probiotic intervention with *Lactobacillus para casei* LPC-37, *Lactobacillus rhamnosus* HN001, *Lactobacillus acidophilus* NCFM, and *Bifidobacterium lactis* HN019.^13^ In contrast, two other studies, one in self-reported insomniacs (*n* = 40) with *Lactobacillus plantarum* PS128,^36^ and another involving healthy participants (non-sportive and football players, *n* = 27) taking *Bifidobacterium lactis* CBP-001010, *Lactobacillus rhamnosus* CNCM I-4036, and *Bifidobacterium longum* ES1,^37^ did not find alterations of VN function after probiotic therapy. Notably, the latter two studies had very low sample sizes. We confirmed our earlier cross-sectional findings of a correlation of bacterial diversity and VN function,^9^ which was also shown in a study by Tsubokawa et al .^38^

Our study is the first to investigate HRV alterations in MD patients after probiotic therapy. Research has shown that only certain bacterial species may have an impact on VN function. For example, some species such as *Lactobacillus plantarum, Bacillus subtilis, Escherichia coli, and Staphylococcus aureus* can produce acetylcholine, possibly acting as a VN stimulant.^39^ Notably, significant differences could be found only after three months of probiotic intake for autonomic parameters, underlining the necessity of long-term intake. Although fast transmission through neuropods has been proposed through the VN,^40^ probiotics may take longer to significantly affect the ANS, as rapid neural transmission through the VN has been recently questioned and slower paracrine activation of VN fibers may be more prevalent.^41^ Interestingly, vagus nerve stimulation for treatment resistant depression also takes several months to develop significant clinical effects.^42^ Thus, long-term application of probiotics (more than 3 months) may be necessary to observe significant effects on the VN. This observation aligns with improvements seen in other conditions, such as irritable bowel syndrome and nonalcoholic fatty liver disease which require extended therapy durations to achieve noticeable benefits.^43–45^ Additionally, a study using the same probiotic as in our study, was only able to show significant differences in depression outcomes after 6 months of treatment in patients with post-infectious fatigue.^46^

Interestingly, VN function changes were observed mainly in the morning, not in the afternoon or night, possibly due to probiotics balancing the nervous system in particularly stressful times of the day. The body experiences higher sympathetic activation and stress levels in the morning due to the release of cortisol during the cortisol awakening response (CAR). So far, some studies described changes in CAR following a probiotic and prebiotic intervention, while others did not, which may be related to differences in measurement techniques.^36,47,48^ Also, predominant effects of the probiotic in the morning could be due to medication effects. Many antidepressants have been shown to affect the gut microbiome and its composition^49^ as well as affecting sleep architecture.^50^ Another possibility could be that there is a change in HRV in the morning due to a cumulative effect of improving sleep quality and taking probiotics in patients with depressive illness.^51,52^ In line with previous studies in HC,^53,54^ sleep was significantly improving in MD participants in the probiotic group, which is important because sleep disturbances represent a core symptom in MD.^55^

Our findings confirm previous studies demonstrating distinct gut microbiome differences between patients and HC.^56,57^ Specifically, *Lactococcus lact*is, included in our study’s probiotic, was found to be more prevalent in HC, but not in MD, following probiotic intake. The gut microbiome’s complexity suggests that probiotics can induce significant effects on gut function and host health, even if the administered species are not directly detectable in stool samples. After three months of probiotic intake, MD participants who were in the probiotic group had significantly higher abundance of Christensellales, particularly *Akkermansia muciniphila*. *Akkermansia muciniphila* is a gram-negative, mucin-degrading bacterium that resides in the mucus layer of the human gut. It plays a crucial role in maintaining gut health by modulating the gut barrier, immune responses, and metabolic processes. The abundance of *A. muciniphila* is often inversely correlated with neuropsychiatric states, suggesting its role as a beneficial microbe in promoting intestinal homeostasis and overall health.^58^ A recent study highlighted the role of *Akkermansia* as a possible treatment for depression as it improved depressive like symptoms and modulated 5-HT levels in the gut and brain of mice.^59^

Consistent with our previous study,^60^ the probiotic intervention did not significantly affect depression scores, probably due to a large placebo effect, which is also common in psychopharmacological studies.^61^ Regarding the LEIDS-r questionnaires, there was a trend to reduced rumination in depressive subjects taking the probiotic, similar to our earlier findings in individuals with euthymic bipolar disorder.^62^ Further, as seen in prior studies in HC,^53,54^ sleep was significantly improved in MD participants in the probiotic group, which is crucial given that sleep disturbances are a core symptom in MD.^55^

This study has some limitations: First, VN function and the gut microbiome can be influenced by several factors including diet, relaxation techniques, sleep and physical activity. To mitigate this, we used 24-hour measurements for long-term VN function and advised participants to maintain their usual routines. Futher, the recruitment of this study was undertaken during the COVID-pandemic, which may have affected the stress levels; however, the stress levels of the probiotic group and placebo group did not differ significantly. Notably, depressed patients continued their usual antidepressant treatments, which can affect gut microbiome composition and HRV measurements, as described in previous studies.^63,64^ Probiotics in this study were intended solely as an add-on therapy, in line with current recommendations,^65^ and not as a replacement for standard pharmacological treatment. While both the probiotic and placebo groups received standard pharmacological care equally, minimizing differential effects of medication, the impact of ongoing antidepressant use on our findings cannot be fully excluded. This factor should be considered when interpreting the results. Another limitation of this study is that, despite efforts to ensure compliance through instructions, weekly check-ins, and the collection of unused capsules, many patients did not return their sachets, resulting in incomplete adherence data. As is typical in naturalistic studies, full compliance cannot be ensured, and this may impact the accuracy of intervention adherence.

Further, in the depression group there were significantly more smokers, which could also influence the gut microbiome and VN function, leading to an increase in sympathetic drive and a decrease in HRV.^66,67^ The relatively strong decrease of depressive symptoms over time is consistent with the expectation that treatment as usual would be effective for treating mood symptoms.^61^

This study suggests that adding probiotics to standard therapy may help balance microbiota in individuals with MD, improving VN function and sleep, though long-term use may be necessary. Further research should examine how targeted psychobiotics^68^ affect VN function in psychiatric disorders and how the observed effects change over time.

In conclusion, depression remains a multifactorial disorder that requires a multifactorial treatment approach. Broadening current treatment plans of MD to include lifestyle changes as a foundational component,^69^ including the addition of a probiotic,^10^ preferably containing species which have been shown to influence the VN, may improve patient outcomes.

## Data Availability

The data that support the findings of this study are openly available in Zenodo, reference number 10.5281/zenodo.13885184.
